# Economic vulnerability to tropical storms on the southeastern coast of Africa

**DOI:** 10.4102/jamba.v12i1.676

**Published:** 2020-10-19

**Authors:** Ernest L. Molua, Robert O. Mendelsohn, Ajapnwa Akamin

**Affiliations:** 1 Department of Agricultural Economics and Agribusiness, Faculty of Agriculture and Veterinary Medicine, University of Buea, Buea, Cameroon; 2 Yale School of the Environment, Yale University, New Haven, Connecticut, United States

**Keywords:** climate change, tropical storms, vulnerability, damage costs, South-Eastern Africa

## Abstract

Climate change will hit Africa economically hard, not least Southeast Africa. Understanding the impact of extreme climatic events is important for both economic development and climate change policy. Global climatological summaries reveal high damage potential pathways for developed countries. Will countries in Africa, especially in the southeastern board of the continent, be vulnerable to loss-generating extreme climate events? This study examined for countries in the sub-region, their vulnerability and damage costs, the impact of climate change on tropical storm damage, as well as the differential impacts of storm damages. An approach using a combination of physical and economic reasoning, as well as results of previous studies, reveals that in Southeast Africa, the economic response to the key damage parameters of intensity, size and wind speed is significant for all the countries. Damages in Kenya and Tanzania are sensitive to wind speed. Both vulnerability and adaptation are important for Madagascar and Mozambique – two countries predicted to be persistently damaged by tropical storms. For Mauritius and South Africa, inflictions from extreme events are expected to be impactful, and would require resilient public and private infrastructure. Reducing the physical and socio-economic vulnerability to extreme events will require addressing the underlying socio-economic drivers, as well as developing critical public infrastructure.

## Introduction

Global warming is already affecting sub-Saharan Africa and it is expected to worsen in coming decades (IPBES 2018; IPCC 2019; UNDP 2019). The region’s present environmental challenges mirror its paleoclimatological past with geological features, providing useful clues of significant diversity of past climate change (Hoelzmann et al. 1998). Although the events varied in their duration, rapidity, spatial extent and climate impacts, Africa’s climatic change has been notable since the last glacial period, when there was a warmer climate around much of the world (Johnson & Odada 1996; eds. Miller et al. 2009). In Africa, there were heavier monsoon rains spreading northward into the Sahara, watering a Saharan steppe which in 5000 years is today’s well-known sandy desert (Goliger & Retief 2007; Thompson et al. 2002). This gradual climate change has had profound implications over the centuries on both human and environmental ecosystems. Moreover, future climate change may alter the frequency and severity of historical events (Goliger & Retief 2007; IPCC 2014; Price, Yair & Asfur 2007; Salinger 2005).

The associated extreme weather events such as droughts and floods have potentially damaging implications for developing countries in Africa (Mugambiwa & Tirivangasi 2017; Pauw et al. 2011). These concerns were particularly reinforced in the previous century, following crises in the 1968–1972 droughts in the Sahel, Ethiopia’s famines of the 1970s and the 1980s (McCann 1999), to name a few. Storm-related disasters have become more frequent especially during the last two decades, from the Atlantic through the Pacific to the Indian basins (Emanuel 2005; Emanuel, Sundararajan & William 2008). The high-wind tropical storms reaching the East African coast and the resulting floods have a particularly devastating impact on peoples’ livelihoods (Goliger & Retief, 2007; Otto et al. 2015; Zwane 2019). Contemporary storms and floods in East Africa include Cyclone Gretelle of 1997, the widespread floods associated with the cyclones Leon-Elyne, Gloria and Hudah of 1998–2000, Idai and Kenneth of 2019. Whilst the extent of the damage varied from country to country, some areas were severely affected (Jordaan, Bahta & Phatudi-Mphahlele 2019; Kusangaya et al. 2014; Mucherera & Mavhura 2020; Munyai, Musyoki & Nethengwe 2019; Zwane 2019).

**FIGURE 1:**
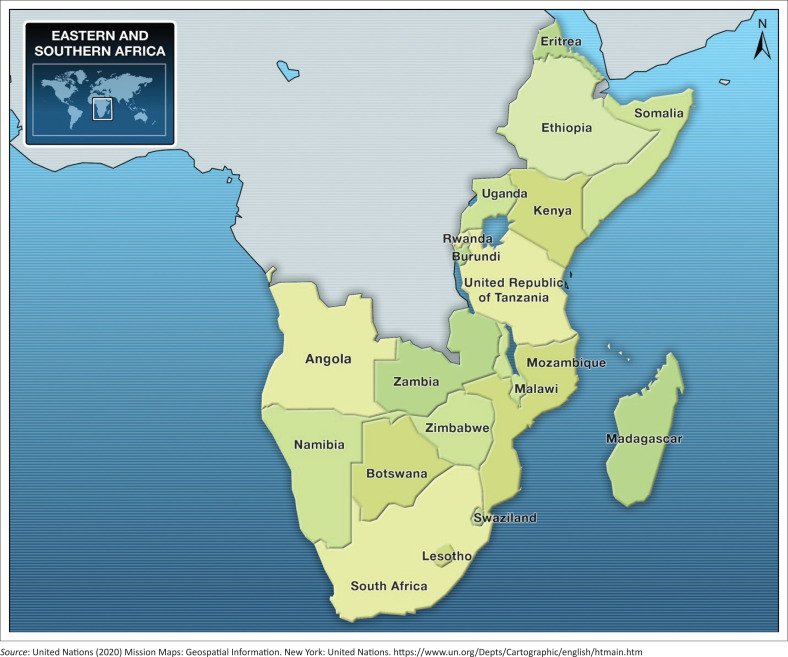
Map of Southeast Africa.

Countries in Southern Africa including its eastern flank, such as Madagascar, Malawi, Mozambique, Tanzania, Zimbabwe and north-eastern South Africa (as shown in Figure 1), experience not only heightened spatial variations of rainfall but also severe droughts and floods. United Nations Economic Commission for Africa (2012) notes that:

[*R*]ural economies in Southern Africa are particularly sensitive to the direct impacts of climate change, because many of them depend heavily on agriculture and ecosystems and because of their high poverty levels and geographic exposure. (p. 2)

According to the Food and Agriculture Organisation (FAO), Mozambique experienced agricultural production losses of 98 000 tonnes of cereals and beans, and the direct and indirect economic damage has been estimated at $1 billion because of climatic events (FAO 2001). As the FAO further notes, in Madagascar, 142 000 of paddy fields, 5000 of maize, 2400 of cassava and 33 000 hectares of export crops were affected. Other similarly affected countries in the region were Botswana, South Africa, Swaziland, Zimbabwe and Zambia. Arndt et al. (2011) note that Mozambique, like many African countries, is already highly susceptible to climate variability and extreme weather events. Climate change threatens to further heighten this vulnerability. For Mozambique, future climate change scenarios show potential damages if there are no changes in policy to be significant, with discounted estimates that range from $2.3bn to $7.4bn during 2003–2050 (Arndt et al. 2011). Food and Agriculture Organisation (2016:3) notes definitively that, ‘… climate change already affects agriculture and food security and, without urgent action, will put millions of people at risk of hunger and poverty…’ and that ‘…while impacts on agricultural yields and livelihoods will vary across countries and regions, they will become increasingly adverse over time and potentially catastrophic in some areas …’

There are therefore country-level and sub-regional experiences that reveal that climate change is undoubtedly one of the biggest crises that humanity is facing today. In Southern Africa, according to Kusangaya et al. (2014), climate change is likely to affect nearly every aspect of human well-being, from agricultural productivity and energy use to flood control, municipal and industrial water supply to wildlife management, as the region is characterised by highly spatial and temporally variable rainfall and, in some cases, scarce water resources. Low adaptive capacity and poverty in the region only increase vulnerability to climate change. According to Mpandeli et al. (2018), Southern Africa is witnessing an increased frequency and intensity in climate-change-associated extreme weather events, causing water, food and energy insecurity. A projected 20% reduction in annual rainfall by 2080 in Southern Africa will only worsen socio-economic conditions in this part of the continent. This may exacerbate regional resource scarcities and vulnerabilities. It will also have direct and indirect implications for human nutrition, health and overall well-being. Reduced agricultural production, lack of access to clean water, sanitation and sustainable energy are the major areas of concern. The region is already experiencing an upsurge of vector-borne diseases (malaria and dengue fever), and water- and food-borne diseases (cholera and diarrhoea). What is clear is that climate change impacts are cross-sectoral and multidimensional, and therefore, require cross-sectoral mitigation and adaptation approaches. Recently, Zwane (2019) assessed the impact of climate change on primary agriculture and food security in South Africa and noted that many dams had water levels as low as 40% during 2016/2017; this led to a reduction in yields for crops such as grapes. Droughts have become recurrent, affecting both small-scale and commercial farmers. The beef and dairy industry are not spared as livestock production has suffered a decline over time. These are unfolding disasters requiring sustained relief efforts. According to Mucherera and Mavhura (2020), disasters result from the interactions of hazards and vulnerability conditions. For example, they explored the perspectives of survivors of floods in Zimbabwe on vulnerability to floods and showed that shortage of land, flood-based farming practices, poverty and climate change, amongst others, are the key factors that increase vulnerability to floods amongst smallholders. The most affected groups of people are often women, children and the elderly.

The problematic surrounding extreme events are timely experienced by all stakeholders – households, communities or nations – as unfolding disasters are beamed into living rooms by an omnipresent media. Hill and Nhamire (2019) writing for Bloomberg news reported that the worst hurricane in a decade killed dozens in Southern Africa. According to BBC reports, Cyclone Idai made landfall near the port city of Beira in Mozambique’s Sofala province mid-March 2019, packing winds of up to 177 km/h and bringing torrential rain which swept through Mozambique, Malawi and Zimbabwe, destroying towns and villages in its path (BBC 2019):

Hundreds of people were killed and hundreds of thousands more affected by what the UN said could be ‘one of the worst weather-related disasters ever to hit the southern hemisphere’. (n.p.)

At least 43 people died in central Mozambique and Zimbabwe after a tropical cyclone tore through the southern African nations, knocking out electricity and phone networks and cutting power to South Africa from a hydropower dam. (Hill & Nhamire 2019)

The apt description of the event provides succinct anecdotal evidence on the damage wrought by environmental factors. According to Hill and Nhamire, Cyclone Idai that had wind speeds of more than 200 km/h before it made landfall exacerbated flooding in the region, which killed more than 60 people. In some years, the events unfold in quick succession, exposing the inadequacy of communal, national and regional response.

Concerns are therefore mounting over increased frequency and severity of these tropical storms and the vulnerability of countries in Southeast Africa to climate change. Climate change is likely to alter the distribution of climatic hazards, which are locally important in terms of human lives and property, and general economic performance (Salami, Von Meding & Giggins 2017). Globally, households in the coastal areas would be more vulnerable to storms and floods inducing damages to life and property (Dasgupta et al. 2009; UNDP 2007). In the face of these challenges to Africa’s productive capacity, the goal of this article is to examine the expected welfare loss from climate-change-induced tropical storms. Specifically, the article reviews the effects of climate change and tropical storms, and assesses the vulnerability and damage that have been inflicted on the Southeast coast of Africa since the 1960s, and makes further projections. Estimating and discussing how these impacts vary across countries in the region and how damages are distributed across hurricanes is important as it contributes to the understanding and the reducing of the vulnerability to extreme events, which requires such information for current development objectives. This assessment is also important, providing directions on alternative approaches for lowering the costs for disaster reduction, which features prominently in the UN Framework Convention on Climate Change Bali Action Plan (UNFCCC 2007). To achieve the goal of the study, the rest of the article is structured into four subsections. Section ‘The Nexus of tropical storms and climate change’ x-rays the nexus of tropical storms and climate change. Section ‘Materials and methods’ highlights the methodology employed. Next, we present section ‘Results and discussion’. The article ends with some recommendations and concluding comments in the last section.

## The nexus of tropical storms and climate change

Whilst the scientific link between tropical storms and climate change is being established, increases in average global temperatures are likely to result in enhanced precipitation, atmospheric moisture and circulation (IPCC 2014). Even with uncertainties in early Atlantic hurricane records, a connection has been shown between sea surface warming and hurricane activity (Mann et al. 2007; Webster et al. 2005). This is further linked with the unusually warm sea surface temperatures, for instance, the Atlantic hurricane season (Trenberth & Shea 2006). This confirms the correlation observed between sea surface temperatures and a hurricane’s destructive potential (Emanuel 2005). This, in turn, leads to more severe tropical storms and hurricanes (IPCC 2007, 2014). Knutson et al. (2010) note that whilst large amplitude fluctuations in the frequency and intensity of tropical cyclones complicate both the detection of long-term trends and their attribution to rising levels of atmospheric greenhouse gases, future projections based on theory and high-resolution dynamical models consistently indicate that greenhouse warming will cause the globally averaged intensity of tropical cyclones to shift towards stronger storms, with intensity increasing between 2% and 11% by 2100. Some studies, however, project decreases in the globally averaged frequency of tropical cyclones by 6% – 34% (Karl et al. 2008; Nordhaus 2010; Pielke et al. 2008; Vecchi, Swanson & Soden 2008). Balanced against this, certain higher resolution modelling studies typically project considerable increases in the frequency of the most intense cyclones, and increases of the order of 20% in the precipitation rate within 100 km of the storm centre (Emanuel 2007; Knutson et al. 2010; WMO 2006). In addition to the intensity of the hurricane, the frequency is also noted to increase by 40% with a 0.5 °C rise in sea surface temperature (Trenberth & Shea 2006).

Consequently, on this premise, climate change is expected to result in increased storm frequency (Banholzer Donner, 2014; IPCC 2014; Keim & Robins 2006; Price et al. 2007). High-intensity storms may culminate in natural disasters, which are a threat to progress everywhere in the world. Countries in Southeast Africa are characterised by significant climatic variability (Joubert, Mason & Galpin 1996). Some South African studies have indicated the possibility of extreme occurrences related to climate change (Howard et al. 2019; Joubert et al. 1996; Mather & Stretch 2012; Williams, Kniveton & Layberry 2011). These include increases in the amount of rainfall (Mason et al. 1999) and significantly high temperatures (New et al. 2006). Varying trends in precipitation in the Southeast including Botswana and Zambia are recorded (Shongwe et al. 2009). Further, the number of disasters is on the rise according to the International Emergency Disasters Database (EM-DAT) (Shongwe et al. 2009), with the average number of reported natural disasters in the region rising from five a year in the 1980s to over 18 annually in the period 2000–2006. East Africa, which received increased precipitation, suffered increased flooding, damage to infrastructure and ecosystems. This means that meagre resources that would otherwise be earmarked for development projects were diverted to relief aid and reconstruction. Increased frequency of tropical storms is therefore likely to exacerbate management problems relating to public health, infrastructure and production (Lesk et al. 2016; Mpandeli et al. 2018; Nhamo & Muchuru 2019; Salami et al. 2017)

Past experiences on the effects of disasters provide an indication of the future costs of such disasters. The damage because of floods in Mozambique caused by Tropical Storms Elyne and Gloria in February and March 2000 was estimated at $1bn, compared with the country’s export earning of only $300 million in 1999. This is an important indication of the high level of economic cost to the country, given the increased frequency and intensity of such storms. Increased population pressures, effects of poverty and other environmental changes may exacerbate these effects. In turn, the associated extreme weather events may severely undermine economic growth and poverty reduction, especially in food-insecure, low-income countries (Mucherera & Mavhura 2020; Munyai et al. 2019; Zwane 2019). Such events usually have economy-wide implications beyond directly affected sectors or regions, as production chains are disrupted, assets depreciate and consumer demand declines (Abidoye & Odusola 2015; Mugambiwa & Tirivangasi 2017; Nhamo & Muchuru 2019; Van Der Veen 2004).

A number of studies have estimated the economy-wide losses occurring during extreme events (e.g. Arndt & Bacou 2000; Boyd & Ibarrarán 2008; Horridge, Maddan & Wittwer 2005; Lesk et al. 2016; Narayan 2003). Lesk et al. (2016) estimate national cereal production losses across the globe resulting from reported extreme weather disasters during 1964–2007 and show that droughts and extreme heat significantly reduced national cereal production by 9% – 10%. On analysing the underlying processes, Lesk et al. (2016) find that production losses because of droughts were associated with a reduction in both harvested area and yields, whereas extreme heat mainly decreased cereal yields. Within southeastern Africa, Pauw et al. (2011) estimate that at least 1.7% of Malawi’s gross domestic product (GDP) may be lost each year because of the combined effects of droughts and floods. Whilst smaller scale farmers in the southern region of the country could be worst affected; however, poverty amongst urban and nonfarm households may also increase because of nationwide food shortages and higher domestic prices. Nordhaus (2006) predicts that hurricane damages would therefore double by 2100 an increase of 0.06% of GDP. Narita, Tol and Anthoff (2008) estimate that global hurricane damages would increase by 0.007% a year. Bouwer and Botzen (2011) and Nordhaus (2010) assert the substantial vulnerabilities to intense hurricanes in the Atlantic coastal United States and vouch that future climate change may impact on hurricane damage; they, however, disagree on the extent of the damages. Nordhaus (2010) reveals that the average annual US hurricane damages will increase by $10bn or 0.08% of GDP because of global warming.

For island states such as Madagascar, Sychelles and Mauritius in the Indian Ocean, geographic predisposition reinforces their vulnerability. However, for mainland states, this is further reinforced, *inter alia*, by socio-economic factors such as heavy reliance on rain-fed agriculture for income and employment (Mugambiwa & Tirivangasi 2017; Zwane 2019). In addition to potential damage to infrastructure such as roads, public and commercial buildings and housing because of natural disasters, low-lying areas may have a profound and adverse impact on the poor (see, e.g. Goliger & Retief 2007; Heltberg, Jorgensen & Siegel 2008; Morton 2007; Raleigh, Jordan & Salehyan 2008; Reuveny 2007). However, Hertel and Rosch (2010) note that agriculture is the primary means by which the impacts of climate change are transmitted to the poor and it is a sector required in the forefront of climate change mitigation efforts in developing countries. This is true for sub-Saharan Africa where the majority of the poor live in rural areas where agriculture is the predominant form of economic activity, with their fate inextricably interwoven with that of farming (Christiaensen, Demery & Kuhl 2006). Paradoxically, agriculture in the region and in most of the tropics is one of the sectors that are most vulnerable to climate change (FAO 2016; Fischer, Shah & Van Velthuizen 2002; Müller 2001; World Bank 2010). Of all major world regions, Africa is projected to rank highest in yield reductions (FAO 2016; Thornton et al. 2009). In cases of huge food losses because of extreme weather events, the poor at the frontline are particularly vulnerable to increases in food prices, as demonstrated by the poverty consequences of food price spikes (De Hoyos & Medvedev 2009; Ivanic & Martin 2008; Kompas, Pham & Che 2018). Hence, for policy reinforcement of adaptive capacity, there is need for information not only on the determinants but also on the potential magnitude of damage across different sectors because of tropical rainstorms.

## Materials and methods

### Study area

The countries selected for this study are either members of the Southern African Development Community (Mauritius, South Africa and Mozambique) or the East African Development Community (Kenya and Tanzania). The economies of these countries are at different stages of development. Kenya has developed a market-based economy with a liberalised external trade system and few state enterprises. These enterprises are in diverse sectors of agriculture, forestry, fishing, mining, manufacturing, energy, tourism and financial services. As of 2019, Kenya had an estimated GDP of $99.25bn and per capita GDP of $2010. With a GDP of about $41.33bn, the economy of Tanzania is the second largest in the East African Community. However, the country is largely dependent on agriculture for employment, accounting for about half of the employed workforce. The Island of Madagascar has a market economy supported by its well-established agricultural industry and emerging tourism, textile and mining industries. Mauritius has a mixed developing economy based on agriculture, exports, financial services and tourism. Both public and private enterprises have invested heavily in these subsectors, including real estate. Mozambique’s checkered history of a brutal civil war means that the country’s long-term recovery makes it dependent upon foreign assistance. Agriculture employs more than 80% of the labour force and provides livelihood to the vast majority of its inhabitants, mostly in the sub-sectors of fish, timber, cashew nuts and citrus, cotton, coconuts, tea and tobacco. South Africa has the most industrialised and diversified economy in the continent. Being an upper-middle-income economy, both state-owned and private enterprises play a significant role in the country’s economy. South Africa’s membership in the G20 and BRICS group of economies is testament to its economic resilience and developmental progress.

On average, for countries in the Southeast Africa, agriculture plays a major role in the economy, employing a significant population in the region. About 70% of the region’s population depends on agriculture for food, income and employment. However, with the exception of South Africa, much of this agriculture is subsistence farming rather than large-scale production of high-value crops for export. Meanwhile, mining employs almost 5% of the population but contributes 60% of the foreign exchange earnings and 10% of GDP. Tourism is also a rapidly growing industry. Although life expectancy is improving, there are significant variations across the region. Mauritius and the Seychelles continue to have the highest life expectancy at 73 years, whereas the lowest life expectancy is found in Lesotho at only 46.7 years. The average life expectancy in Southeast Africa is 53 years. About 25% of the population is urban. These countries are vulnerable to a range of natural disasters. Since 2000, countries in Southern Africa have experienced an increase in the frequency, magnitude and impact of drought and flood events. Climate change is expected to significantly affect the region and increase risks related to water resources, fire, agriculture and food security. Furthermore, island states such as Madagascar and Mauritius have their own unique set of problems – climate change has left the countries in danger of losing protective reef barrier and a sea-level rise could threaten its survival.

### Analytical model

This article views storm as a generic term to describe a large variety of atmospheric disturbances ranging from ordinary rain showers to thunderstorms, wind and wind-related disturbances such as tornadoes, tropical cyclones and sandstorms. The theoretical foundation based on Bakkensen and Mendelsohn (2016, 2019) and Mendelsohn et al. (2012) assumes that the economic damage (*D*) from each storm is the sum of all the losses caused by it. Damages could be lost buildings, infrastructure and human fatalities. However, damages of buildings and infrastructure provide for better records. The economic damage of capital losses is the present value of lost future rents and this should be equal to the market value of the building. We note that the market value of capital is often less than the replacement cost.

Expected damages of hurricane with particular characteristics (*X*) are the frequency or probability (*π*) the hurricane will occur in each place, with damages depending upon where the hurricane strikes (*i*). Important characteristics of the hurricane include minimum barometric pressure (*MP*) and maximum wind speed (*WS*). A hurricane (*j*) with particular characteristics strikes each place (*i*) given the climate (*C*), and has probability of occurrence

$$\pi_{ij}=\pi (X_{ij},C)$$ [Eqn 1]

The actual damages associated with any given hurricane (*j*) also depend on the vulnerability (*Z*) of each place (*i*). Vulnerability concerns the susceptibility of society to substantial damage, disruption and casualties as a result of a hazardous event (OECD 1994). Smit et al. (1999) view vulnerability as the ‘degree to which a system is susceptible to injury, damage, or harm (one part – the problematic or detrimental part – of sensitivity)’. According to Brooks, Adger and Kelly (2005), vulnerability depends critically on context, and the factors that make a system vulnerable to a hazard will depend on the nature of the system and the type of hazard in question. Therefore, a hurricane will only turn into a disaster if it strikes a populated area with infrastructure or crops. In other words, most storm-related disasters will occur in regions that often do not have the wealth, infrastructure and institutional capacity to protect their people against tropical storms. Assuming, for example, that the damage function in each location (*i*) could depend on population density (*POP*) and income (*Y*):

$$D_{i}=D(X_{i},Z_{i})$$ [Eqn 2]

Actual damages will also depend upon the adaptation (*A*) measures taken to prevent extreme event damage. The expected value of hurricane damages is

$$E[D]=\sum_{j}\sum_{i}\pi (X_{ij},C)D(X_{i},Z_{i})$$ [Eqn 3]

The damage caused by moving from the current climate C0 to a future climate C1 is the change in the expected value of the extreme events:

W=E[D(C1)]−E[D(C0)] [Eqn 4]

This value is summed across all the storm events. For any given time period, damages from climate change are imminent, given the variation in the frequency, intensity or locations of storms. The calculation of hurricane damages is performed for each country. The calculation of the damages with and without climate change is performed holding the characteristics of each country constant. In other words, the analysis firstly compares the damages from tropical storms with the current economic baseline with damages caused with the future economic baseline. Then, the estimates of the impact of tropical storms with the future climate minus the impact of tropical storms with the current climate are analysed to estimate the effect of climate change. ^1^

Weitzman (2010) reiterates the need for functional forms that capture reality adequately, and are analytically sufficiently tractable to yield useful results. The following functional form is therefore employed to estimate regression coefficients that used to predict the damages that would be caused by each storm in the generated data set:

$$\ln\;D_{it}=\alpha_{0}+\alpha_{1}\ln Y_{it}+\alpha_{2}\ln WSP_{it}+\alpha_{3}\ln POP_{it}+\alpha_{4}\ln HDI_{it}+\varepsilon_{i}$$ [Eqn 5]

where *D*_*it*_ is normalised damages for country *i* in year *t, Y*_*it*_ is gross income measured as GDP per capita, *WSP*_*it*_ is the wind speed in m/s, *POP*_*it*_ is the population density, *HDI*_*it*_ is the United Nations Human Development Index (HDI) – a proxy for vulnerability, *α*_*i*_ is the country-specific coefficient and ε_*i*_ is the error disturbance term.

### Projections and data source

This article views storm as a generic term to describe a large variety of atmospheric disturbances ranging from ordinary rain showers to thunderstorms, wind and wind-related disturbances such as tornadoes, tropical cyclones and sandstorms. The economic damage from each storm, obtained from EM-DAT (2019),^2^ is assumed to be the sum of all the losses caused by it. Damages could be lost buildings, infrastructure and human fatalities. However, damages of buildings and infrastructure provide for better records. The economic damage of capital losses refers to the present value of lost future rents. This should be equal to the market value of the building. The analysis is then conducted on major storms striking the region, though this data set has no information about the magnitude of the storm, it allows for the estimation of the coefficients for vulnerability (income and population density). The data on wind speed are obtained from NOAA. In line with Bakkensen and Mendelsohn (2016) and Mendelsohn et al. (2012), a damage function is then used to predict the damages that each storm will cause. The coefficient for storm magnitude was estimated using aggregate damages per storm and storm characteristics at landfall. The projections for future climate change rely on four climate models: National Centre for Meteorological Research (CNRM) (Gueremy et al. 2005), ECHAM (Cubasch et al. 1997), GFDL (Manabe et al. 1991) and MIROC (Hasumi & Emori 2004). The models are used to predict the expected frequency of hurricanes. The model calculated damages for each storm in the data set given its intensity and where it landed. The expected damages were calculated by summing the product of the probability of each storm times the damage it caused. Separate estimates were made for each country. The other data are collated from other sources, for example, information on historic rainfall and temperature is generated from the Africa Rainfall and Temperature Evaluation System (ARTES). Information on national incomes and population is obtained from the PENN World Tables. Information in HDI is collated from the United Nations Development Programme (UNDP).

### Ethical consideration

The authors confirm that ethical clearance was not required for the study.

## Results and discussion

### Threats, vulnerability and damage costs

Analysis of the data shows that Southeast Africa is a region haunted with socio-economic and environmental challenges that are reinforced by inherent vulnerabilities, amongst which is an incessant climatic threat. Using storm information from the Emergency Events Database (EM-DAT 2019) that shows the major storms striking the globe, the aggregate damages per storm and storm characteristics at landfall provide important information on storm magnitude for the regions. Records for the last 100 years show 41 local storms, 99 tropical cyclones and 54 unspecified windstorms making landfall on the African continent (EM-DAT 2019). The high winds that accompany both the local storms and the resulting floods have had a particularly devastating impact per event, affecting on average 9424 persons and killing 22 persons per storm (Table 1). The stronger tropical storms affected 149 267 people, killing 33 persons. The damages incurred have averaged $16.306m for local storms and $31.075m for tropical storms over the last 100 years. The FAO notes that whilst the intensities have hardly changed during the last three decades of the 20th century, their frequency appears to be on the increase (FAO 2001). Furthermore, the devastation caused by tropical storms rose enormously during the 1990s, due in part to the population increase in storm-prone areas. According to the World Disasters Report of the International Federation of the Red Cross (IFRC 2000), wind storms and flood-related disasters during 1990s altogether accounted for 60% of the total economic loss caused by natural disasters. A significant percentage of disaster casualties, in terms of deaths, injuries and people displaced from their homes and livelihoods, were attributable to storms and floods. The years 2000 and 2001 witnessed a huge flooding event in Mozambique, particularly along the Limpopo, Save and Zambezi valleys (FAO 2001; IFRC 2000; IRIN 2010; Reuters 2007). In 2000, floods resulted in half a million people made homeless and 700 losing their lives. The floods had devastating effects on livelihoods, destroying agricultural crops, disrupting electricity supplies and demolishing basic infrastructure such as roads, homes and bridges. The levels of losses in such disasters demonstrate the economic importance of reducing vulnerability.

**TABLE 1:** Storm profile on the African continent, 1900–2019.

Events	Number of events	Number of persons killed	Total persons affected	Damage (000 US$)
Local storm	41	915	386 401	668 563
Average per event	-	22.3	9424.4	16306.4
Tropical cyclone	99	3361	14 777 481	3 076 430
Average per event	-	33.9	149 267.5	31 075.1
Unspecified	54	581	95 480	3725
Average per event	-	10.8	1768.1	69

The observations in Table 1 on persons killed, persons affected and damage incurred reveal heightened vulnerability. Anecdotal evidence in Madagascar and Mozambique provides illumination on the plausibility of vulnerable societies bearing the brunt of climatic extremes (Reuters 2007; The Guardian 2011). According to the Organisation for Economic Cooperation and Development (OECD), vulnerability concerns the susceptibility of society to substantial damage, disruption and casualties as a result of a hazardous event (OECD/DAC 1994). In other words, vulnerability implies the degree to which a system is susceptible to injury, damage or harm (one part – the problematic or detrimental part – of sensitivity) (Smit et al. 1999). However, vulnerability depends critically on context, and the factors that make a system vulnerable to a hazard will depend on the nature of the system and the type of hazard in question (Brooks et al. 2005). These analytical findings are corroborated with field reports. For example, Madagascar was hit by three or four tropical storms in an average year. In 2004, Tropical Cyclone Gafilo with south-westerly winds blowing at 120 km/h and gusts as high as 180 km/h left at least 55 000 people homeless (SAPA 2004). In 2007, Madagascar appealed for $242m to fix seasonal storm damage that affected about 25 000 people and left more than 7000 homeless (Saholiarisoa 2007). In the first few months of 2008, three consecutive cyclones (Fame, Ivan and Jokwe) struck Madagascar affecting 17 of the 22 regions. Accompanied by heavy rainfall, these category 3 and 4 storms caused extensive physical destruction to infrastructure and affected the livelihoods of about 342 000 people. Cyclone Ivan with sustained winds of 111 km/h swept across Madagascar, knocking out power and communication (CNN 2008).

Unlike subtle changes in precipitation and temperature, recorded changes in the frequency and intensity of storms and related events of storm surges and floods are not only perceived but are also unfolding experiences which attract the attention of the media given the hazard-related capacity of these phenomenon. The disruption of life, carnage, loss and damage of extreme weather events is vividly captured in timely reporting by media organisations (CNN 2008; Reuters 2007; The Guardian 2011 ). Reports indicate that:

[*I*]n January 2011, flooding in South Africa killed more than 100 people, forced at least 8400 from their homes and prompted the government to declare 33 disaster areas. During this period, almost every country in southern Africa was on alert for potentially disastrous flooding. In South Africa, 88 deaths were in the eastern KwaZulu-Natal province. The costs of damage to the infrastructure in the seven of the country’s nine provinces affected was estimated at 160 billion rand. The Johannesburg area and northern and eastern provinces experienced some of their greatest rainfall in 20 years. Flimsy houses in townships, where drainage systems are typically poor, were particularly vulnerable to the deluge, affecting about 20 000 people, or about 5000 families, requiring humanitarian assistance. (n.p.)

In Table 2, we present the results of the historical damage functions. The results suggest that damages are sensitive to wind speed. Damages increase by 2.8% and 1.95% for Kenya and Tanzania, respectively, for every 1 m/s increase in wind speed. For the island states of Madagascar and Mauritius, wind speed accounts for 7.3% and 8.2% of damages, respectively. When population change interacts with wind speed, damages rise by 5.8% and 3.3% for Mozambique and South Africa, respectively, for every 1 m/s increase in wind speed. Even experiencing low-intensity storms in countries such as Tanzania, the damages are still significant. Burrus et al. (2002) argue that whilst low-intensity hurricanes typically cause far less structural damage than high-intensity hurricanes, these weaker hurricanes do impact regional economic activity through business interruption, and the cumulative impact of frequent business interruption may be significant through its corresponding effects on employment, business taxes, utility service, employee absenteeism, supply chain interruption and disruption of consumer access to businesses because of temporary flooding, and so on. Hence, low-intensity storms raise the possibility that low-intensity hurricanes may have a significant impact on regional economies over time.

**TABLE 2:** Estimates of historical damage functions.

Variable/Statistic	Kenya	Madagascar	Mauritius	Mozambique	South Africa	Tanzania
Log(Income)	−0.238 (−2.695)*	−0.351 (−7.199)*	−0.169 (−3.334)*	−0.478 (−2.337)	−0.188 (−2.322)	−0.459 (−3.554)*
Log(Population density)	0.144 (1.934)	0.625 (2.382)	0.506 (1.964)	0.395 (3.641)*	0.193 (2.173)	0.132 (3.041)*
Log(Wind speed)	0.281 (1.891)	0.731 (2.974)*	0.823 (3.689)*	0.637 (2.565)*	0.319 (1.586)	0.195 (1.815)
Log(HDI)	−0.094 (−1.976)	−0.394 (−2.267)	−0.255 (−1.962)	−0.028 (−1.986)	−0.153 (−1.981)	−0.039 (−2.611)
Log(HDI)^2^	−0.145 (−2.274)	−0.321 (−2.639)*	−0.287 (−2.654)*	−0.283 (−2.166)	−0.261 (−2.163)	−0.222 (−3.681)*
Log(HDI × Number of disasters)	−0.032 (−1.987)	−0.053 (−1.653)	−0.043 (−1.751)	−0.051 (−1.524)	−0.016 (−1.711)	−0.022 (−1.692)
Log(Population density × Wind speed)	0.521 (2.692)*	0.631 (2.867)*	0.722 (2.623)*	−0.582 (3.465)*	0.327 (2.169)	0.445 (1.967)
Log (Temperature × precipitation)^2^	0.473 (2.961)*	0.316 (2.469)*	0.328 (1.969)	0.529 (1.953)	0.209 (1.678)	0.272 (2.614)*
Constant	7.561 (6.752)*	46.693 (8.913)*	23.592 (7.468)*	18.864 (6.511)*	15.177 (5.566)*	12.483 (7.722)*
Number of observations	24	24	24	24	24	24
Adjusted *R*^−2^	0.452	0.538	0.517	0.569	0.378	−0.483
*F*-Stat	57.534	138.492	76.618	121.227	89.493	69.671

Notes: The functional form of the regression is log–log as noted in Eqn 4. The *t* statistics are in parentheses. As HDI occupies the range of 0–1, all logged HDI values were negative, whereas the squares of these values were positive. A coefficient of 0.281 for *wind speed* means that damages increase 2.81% for every 1 m/s increase in *wind speed*. A coefficient of 0.521 for *population density x wind speed* means that for a 1% increase in *population density*, damages rise an addition of 5.21% for every 1 m/s increase in *wind speed*.

HDI, human development index.

* , *p* < 0.01 level of significance for two-tailed student’s *t* test. One unit of *wind speed* is 1 m/s and 1 unit of *Income* is a 1% change.

The nature and extent of the damages reveal that vulnerability actually matters. Higher incomes lower damages by 1.88% for South Africa and 1.69% for Mauritius. This contrasts with lower income countries such as Mozambique and Tanzania where income lowers damages by 4.78% and 4.59%, respectively. Similarly, Kenya and Tanzania observe an increase in damages by 1.4% and 1.3% for unit changes in their population density, compared with the Island states of Madagascar and Mauritius that observe increase in damages by 6.3% and 5.1%, respectively. The result is in line with the assumptions in the literature that damages are directly proportional to income and population (e.g. Vincent 2007; Wisner et al. 2004). In other words, damages are higher at lower income levels as higher income people apparently take measures to reduce their vulnerability. Rural populations are more sensitive than urban populations to tropical storms. The Government of Madagascar estimated the total damage and losses caused by the three cyclones in 2008 to be $333m (Government of Madagascar 2008). The damage and losses were concentrated in the agriculture, fisheries and livestock sector ($103.0m); the housing and public administration sector ($127.6m); and the transport sector ($45.7m). Given that the housing and agricultural sectors are primordial to livelihoods, the effects of the storms increased the vulnerabilities of large portions of the population. The government of Madagascar estimated the cyclones’ impact to be equivalent to about 4% of GDP, contributing to a decline of 0.3% in the real GDP growth in 2008, and a 38% decline in the current account of the balance of payments, primarily because of a reduction in agriculture exports, an increase on imports of goods and a reduction of tourism services income (Government of Madagascar 2008).

As Brooks et al. (2005) note, however, actual damages would depend on the vulnerability of each community. Relying on the HDI an important socio-economic variable that is composed of per capita income, average education and literacy rates and average life expectancy at birth, it is revealed that damages decrease by 3.9%, 2.55% and 1.53% for Madagascar, Mauritius and South Africa, respectively. When HDI is doubled for these countries, damages decline by 3.2%, 2.9% and 2.6%, respectively. For Kenya, Mozambique and Tanzania with significant lower levels of HDI, their socio-economic potentials contribute to lowering damages by 0.9%, 0.3% and 0.4%, respectively, and even when HDI doubles, damages are lowered by 1.5%, 2.8% and 2.2%, respectively. Madagascar and Mozambique show that for every 1% improvement in HDI, damages decrease by 0.55% and 0.52%, respectively, for every disaster encountered. Though non-linear, these relationships reassert that vulnerability matters. Patt et al. (2010) observed a similar non-linear relationship between HDI and disaster losses. Kellenberg and Mobarak (2008) and De Haen and Hemrich (2007) have shown that countries with medium HDI values experience the highest average losses, whereas countries with high HDI values experience the lowest losses.

### Impact of climate change on tropical storm damage

We assessed the effect of climate change on the extent of tropical storm damages. Table 3 shows the global damages given the current climate and current baseline conditions, on using modelled climatic conditions. As shown, the assessed damages ranged from $1m to $14m per year for Kenya in 2000, and were projected to rise from $2m to $8m by 2100, even if there were no climate change. Madagascar exhibits the biggest damages in today’s climate, amounting to $17m and reaching $139m according to CNRM estimates for current climatic conditions. By 2100, this is projected to a maximum of $81m. Moderate damages are observed for Mauritius, whilst Mozambique and Tanzania with significant primary commodity economies incur maximum damages at $49m and $52m, respectively. South Africa is the most resilient, incurring lower damages. For a country like Mozambique, the inherent politico-economic vulnerabilities are exacerbated by climatic stress, with attendant loss of life and property. In 2002, for instance, a storm struck Mozambique’s port city of Beira, in which 663 houses were partially destroyed and 117 completely destroyed (IRIN 2010). In 2007, heavy rains from a cyclone sparked off more flooding in Mozambique, as the Buzi River in central Sofala province overflowed its banks and forced 140 000 people from their homes. Further south, tourist resort centres bore the full impact of the cyclone’s 270 kph (170 mph) winds. About 36 000 people in the area lost virtually all their possessions. Another series of cyclones then compounded widespread flooding in the southern and central parts of the country, killing 700 people and driving close to half a million from their homes (Reuters 2007). On examining vulnerability to climate change in least developed countries, Patt et al. (2010) note that vulnerability may rise faster in the next two decades than in the three decades thereafter. Whilst positive returns from socio-economic development trends may offset rising climate exposure in the second quarter of the century, it is in this initial quarter that vulnerability will rise most quickly, echoing an urgency for international assistance to finance adaptation.

**TABLE 3:** Current and future global damages from tropical storms with no climate change.

Climate model	Kenya		Madagascar		Mauritius		Mozambique		South Africa		Tanzania	
	2000	2100	2000	2100	2000	2100	2000	2100	2000	2100	2000	2100
CNRM	1	2	139	81	18	15	19	8	-	-	18	7
ECHAM	3	9	67	55	6	4	35	35	1	0	52	36
GFDL	14	15	110	77	5	12	49	38	3	0	20	18
MIROC	8	8	17	54	1	5	15	38	0	0	11	28

*Source*: Computed from the projections in Mendelsohn, R., Emanuel, K., Chonabayashi, S. & Bakkensen, L., 2012, ‘The impact of climate change on global tropical cyclone damage’, *Nature Climate Change* 2, 205–209. https://doi.org/10.1038/nclimate1357

Note: Expected Damages in millions of US$.

CNRM, National Centre for Meteorological Research; ECHAM, European Centre Hamburg Model; GFDL, Geophysical Fluid Dynamics Laboratory; MIROC, Model for Interdisciplinary Research on Climate.

The extent to which climate change influences the behaviour of tropical storms with ensuing damages is captured in Table 4. When climate change is factored to influence the physical nature of storms, Table 4 indicates Madagascar to be the most seriously affected, with hurricane damages projected to reach $36m per year by 2100.^3^ Mozambique, Tanzania and Mauritius are hit by mean annual damages worth $23m, $16m and $4m, respectively. The average of these damages across the four models is $2m per year for Kenya, −$17.3m per year for Madagascar, $2.5m per year for Mauritius, $1m per year for Mozambique, −$1.5m per year for South Africa and −$13m per year for Tanzania. These conservative estimates will mean that Mauritius, Kenya and Mozambique are more vulnerable to tropical storms that make landfall in the southeastern coast of Africa. However, Madagascar’s geographical disposition with long low-lying stretches of coastal areas makes it more susceptible to storm effects. Over 60% of tropical cyclones that develop in the Indian Ocean affect Madagascar, with all the 22 regions of the country at risk. Le Page (2019) and CNN (2008) reported anecdotal evidence, suggesting the vulnerability and extent of recent damages for both Mozambique and Madagascar.

**TABLE 4:** Global hurricane damages caused by climate change in 2100.

Climate model	Kenya	Madagascar	Mauritius	Mozambique	South Africa	Tanzania
CNRM	1	−58	−3	−10	0	−12
ECHAM	7	−13	−3	0	0	−16
GFDL	1	−34	7	−11	−3	−1
MIROC	1	36	4	23	0	16

*Source*: Computed from the projections in Mendelsohn, R., Emanuel, K., Chonabayashi, S. & Bakkensen, L., 2012, ‘The impact of climate change on global tropical cyclone damage’, *Nature Climate Change* 2, 205–209. https://doi.org/10.1038/nclimate1357

Note: Values are in millions of US$ per year based on A1B emission scenario(720 ppm by 2100) and future baseline.

CNRM, National Centre for Meteorological Research; ECHAM, European Centre Hamburg Model; GFDL, Geophysical Fluid Dynamics Laboratory; MIROC, Model for Interdisciplinary Research on Climate.

**FIGURE 2:**
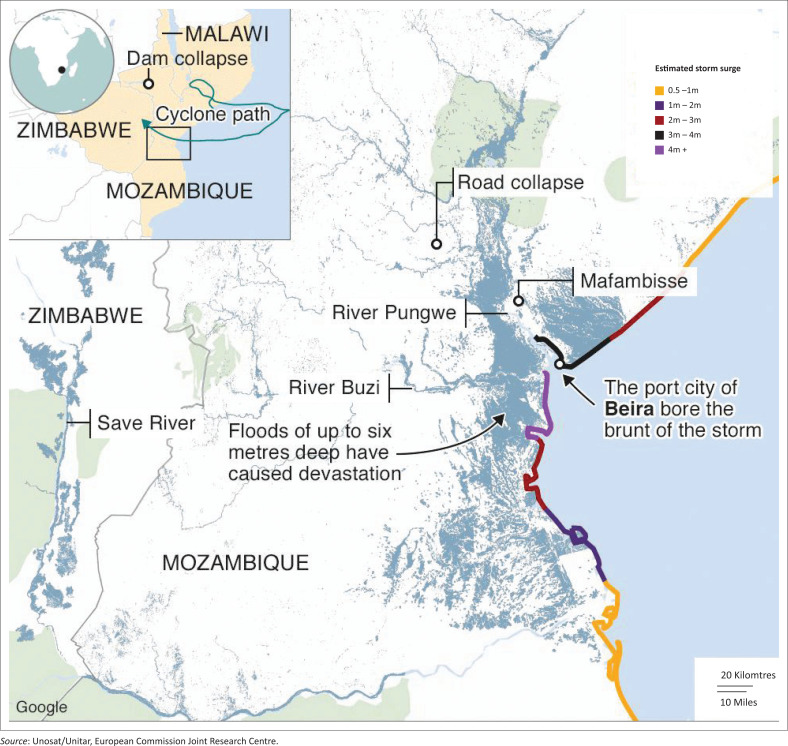
Trail of destruction of Cyclone Idai through Mozambique, Malawi and Zimbabwe.

Hurricane damage distribution is therefore not even across Southeast Africa. Figure 2 describes the damages caused by climate change across six countries in the continent. Madagascar, Mozambique and South Africa are three countries that are projected to be persistently damaged by warming as shown by the MIROC model. The impacts from ECHAM to MIROC are higher because these models are associated with a greater reduction in minimum air pressure. The damages range between $5m for Tanzania and over $35m for Madagascar. Climate change could also trigger off the outbreak and distribution of diseases such as malaria, cholera, Rift-Valley fever and meningitis. The 1997/98 *El-Niño*, for example, was associated with an outbreak of malaria, Rift Valley fever and cholera in many East African countries (FAO 1999).

### Differential impacts of climate change on storm damage

**FIGURE 3:**
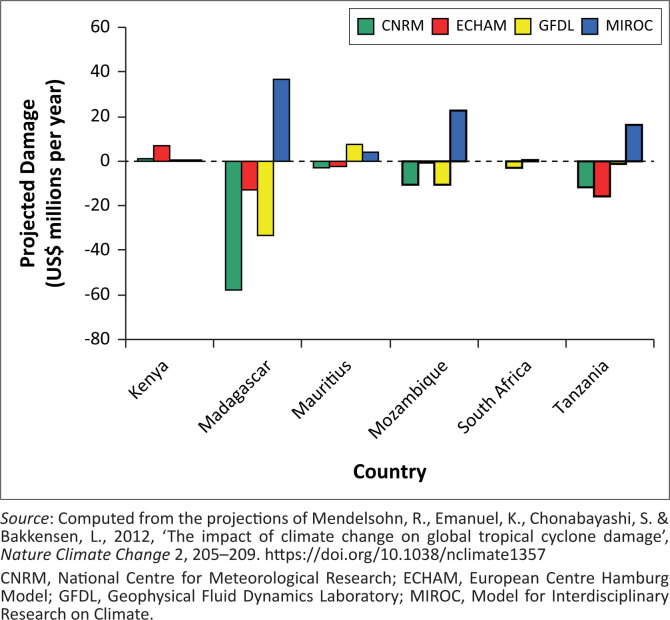
Projected annual hurricane damage by country.

We now evaluate the effect of future climate change on storm damages in the selected countries across the four climate scenarios. We use the predictions of the four climate general circulation models (GCMs): ECHAM (Cubasch et al. 1997), CNRM (Gueremy et al. 2005), GFDL (Manabe et al. 1991) and MIROC (Hasumi & Emori 2004), and estimate the damages for each storm for each country and compare that with current climatic conditions. Climate change may increase the global damages from hurricanes in all four climate scenarios. As shown in Figure 3, projections based on the more pessimistic MIROC model show Madagascar losing almost $38m per year. The more optimistic CNRM model rather indicates resilience for all the countries, except Kenya. On average, however, the global damages worth from hurricanes are expected to increase by $10bn per year, representing a 33% increase, with the biggest and most regular effects in Asia ($4.3bn per year) and North America ($4.8bn per year) (Mendelsohn et al. 2012). The experiences for these African countries are instructive for future policy making to enhance their resilience.

**FIGURE 4:**
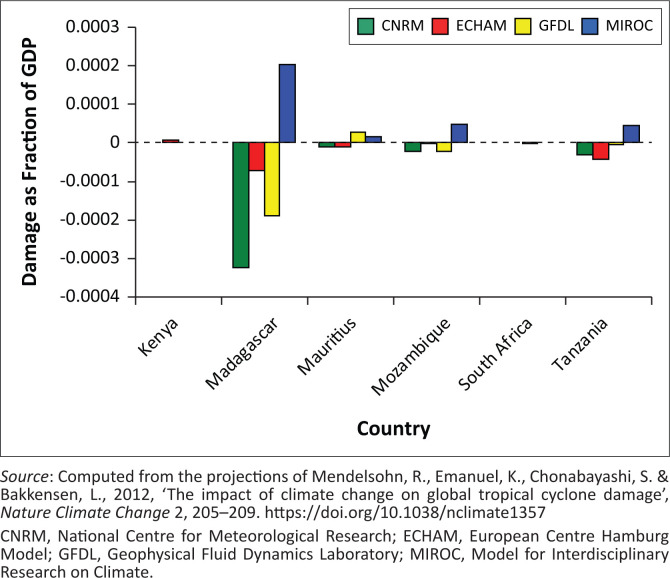
Hurricane damage as a fraction of gross domestic product.

Given the underlying strain on public finances, exogenous climatic shocks will mean further financial strain. Figure 4 displays the projected hurricane damages from climate change as a fraction of GDP in 2100. The figure illustrates how burdensome the change in tropical storm damage will be to the economies in each country. Relying on the MIROC projections, Madagascar has the largest average impact averaging 0.002% of GDP, followed by Mozambique and Tanzania in that order. Although the effects are not consistent across the climate models, Mauritius and Mozambique have the most consistent effects. Madagascar has large average but variable damages. Kenya and South Africa observe little translation of the damages into depletion of their GDP. Overall, these findings project challenges to these African economies, though they are not comparable to the experiences of countries in the Caribbean. For instance, Hurricane Gilbert in 1988 caused Jamaica losses worth 65% of GDP; Hurricane Hugo in 1989 caused Montserrat losses worth up to 200% of GDP; Hurricanes Luis and Marilyn in 1995 caused Antigua and Barbuda losses worth 65% of the GDP (Emanuel 2005; IFRC 2000). This, however, would imply that though minimal, the Southeastern African states need significant economic growth every year to compensate for losses because of climatic disasters.

On examining the average impact on national income from the four climate models, Table 5 shows that damages as a fraction of GDP are expected to fall from their current rate in 2000 for South Africa, Tanzania and Madagascar by 0.00001%, 0.00131% and 0.01627% of GDP in 2100. However, for Mozambique, Kenya and Mauritius, damages are expected to rise by 0.00026%, 0.00057% and 0.00138% of GDP in 2100, respectively. This implies that the countries with the largest average impacts from future climate change are Kenya ($2.2bn) and Mauritius ($1.38bn). Mozambique observes a damage of $0.29bn. Of all the countries that are impacted by hurricanes, the impacts are less than 0.01% of GDP. Though small, this accounts for significant diversion of resources away from meeting developmental goals, and highlights the urgency of transformational processes in developmental planning that empowers and builds resilient communities (Glavovic 2008; Olsson, Folke & Berkes 2004). On reviewing the impact of low-intensity hurricanes on regional economic activity, it is observed that though direct, indirect or induced effects are small, storms produce a cumulative impact that causes significant damages to regional output, regional employment and indirect business taxes (Burrus et al. 2002). Some studies, however, show that the effects of low- and high-intensity hurricanes, for example, loss of output, wealth, jobs and municipal revenues, are more than compensated for by reconstruction, disaster relief programmes and migration, with significant short- and long-run effects (Guimaraes, Hefner & Woodward 1993; Smith & McCarty 1996; West & Lenze 1994; World Bank 2000).

**TABLE 5:** Average impact by country.

Country	Climate Impact (Damage in billion US$ per year)	% GDP
Kenya	2.27	0.00057
Madagascar	−16.93	−0.01627
Mauritius	1.38	0.00138
Mozambique	0.29	0.00026
South Africa	−0.62	−0.00001
Tanzania	−3.12	−0.00131

*Source*: Computed from the projections in Mendelsohn, R., Emanuel, K., Chonabayashi, S. & Bakkensen, L., 2012, ‘The impact of climate change on global tropical cyclone damage’, *Nature Climate Change* 2, 205–209. https://doi.org/10.1038/nclimate1357

GDP, gross domestic product.

Note: Average of results from the four climate models.

These results call for both adaptation and mitigation efforts. Timely adaptation strategies are required to reduce vulnerability to climatic hazards and increase country resilience over the short term (Bakkensen & Mendelsohn 2016; Patt et al. 2010). For instance, Bakkensen and Mendelsohn (2016) find evidence of adaptation being important in most of the world by examining the effects of income, population density and storm frequency on damage and fatalities. According to Patt et al. (2010), promoting adaptation will require that economies take advantage of the relatively narrow policy window over the next decade or so before the impact of climate change becomes significant. Thus, adaptation or adjustment to altered conditions in the face of such events will require a plethora of measures that are inherently interlinked (e.g. institutional, regulatory, technical, biological, behavioural or economic response), but are each required to reinforce the reliance of frontline states in southeastern Africa. Arndt et al. (2011) identify improved road design and agricultural sector investments as key ‘no-regret’ adaptation measures, alongside intensified efforts to develop a more flexible and resilient society. According to Arndt et al. (2011), this could be coupled with cooperative river basin management and the regional coordination of adaptation strategies. Hence, whether it is establishing early warning systems for extreme weather events or coastal protection, education and raising awareness, infrastructure development, environmental planning or perhaps adjustment in land-use planning (housing, forestry and agriculture) and urban development, given recent observations and what we now know, to reduce costs to life and property, adaptation has to be anticipatory rather than reactionary. Schipper (2009) notes that such adaptation is inextricably associated with development, hence a rationale for mainstreaming adaptation into short- and long-term development planning; one of the most effective ways of ensuring that development reduces vulnerability to climate-related hazards.

## Summary and conclusion

Economies of southeastern Africa are already vulnerable to diverse sociopolitical shocks, for which additional strain from environmental challenges such as climate change may lock economic growth with significant macro-economic repercussions throughout the sub-region. Climate change because of global warming is expected to result in an increase in the frequency and severity of tropical rainstorms. This is likely to exacerbate the management problems relating to infrastructure and economic production in vulnerable sub-regions in Africa. This article reviews tropical storm damage that has hit the southeastern coast of Africa since the 1960s and makes projections for the region by 2100. The results suggest that damages are sensitive to wind speed and that vulnerability matters. Further north, damages increase by 2.8% and 1.95% for Kenya and Tanzania, respectively, for every 1 m/s increase in wind speed. And higher incomes lower the damages incurred by 1.88% for South Africa and 1.69% for Mauritius. This contrasts with lower income countries such as Mozambique and Tanzania where income lowers damages by 4.78% and 4.59%, respectively. For Mozambique, Kenya and Mauritius, damages are expected to rise annually by US$ 0.29bn, 2.2bn and 1.38bn, respectively, to the year 2100. Tropical storm damages will therefore cause significant diversion of relatively scarce resources away from development planning for countries in the region.

Whilst the analysis confirms the probable significance of some African states in incurring major damages from climatic extremes, however, the most severe socio-economic and human toll is associated with the smaller, less developed economies, whose capability to rebuild and return to the path of growth and development is limited possibly because of the lack of appropriate institutional response and preventive policies. This is particularly the case of Tanzania and Kenya. These damages may impose additional economic costs at a time when extra resources are needed to finance food, energy and inputs for either the agricultural or manufacturing sectors. The current analysis, though not exhaustive, however, contributes in filling the knowledge gap by providing estimates of potential damage from hurricanes and related storm surges. Given the important societal impacts of tropical storms and the apparent sensitivity of economic factors to tropical climate, further research is strongly recommended to assess the regional sector-related (agriculture, fishery, housing and public infrastructure) effects of these tropical storms.

## Footnotes

1 Otherwise, one will confuse changes caused by economic and population growth with changes caused by climate.

2 The Emergency Events Database (EM-DAT) is provided by the Centre for Research on the Epidemiology of Disasters (CRED) and by the Office of U.S. Foreign Disaster Assistance (OFDA). The Emergency Events Database offers global disaster statistics, including country-level disaster profiles at: www.emdat.be

3 To obtain the impact of climate change, the damages from tropical storms are calculated for the future baseline with and without climate change (Mendelsohn et al. 2012). The future impact of climate change is the difference in damages with a warmer climate and the future baseline minus the current climate and the future baseline. Climate change is measured keeping the baseline constant.
